# Information on the COVID-19 Pandemic in Daily Newspapers’ Front Pages: Case Study of Spain and Italy

**DOI:** 10.3390/ijerph17176330

**Published:** 2020-08-31

**Authors:** Santiago Tejedor, Laura Cervi, Fernanda Tusa, Marta Portales, Margarita Zabotina

**Affiliations:** 1 Department of Journalism and Communication Sciences, Autonomous University of Barcelona, 08193 Bellaterra, Spain; martaportoli@gmail.com; 2 Faculty of Social Sciences, Technical University of Machala, Machala 070201, Ecuador; ftusa@utmachala.edu.ec; 3 International Office, Kazan Federal University, 420008 Kazan, Rusia; MRZabotina@kpfu.ru

**Keywords:** COVID-19, newspapers, media, journalism

## Abstract

Spain and Italy are amongst the European countries where the COVID-19 pandemic has produced its major impact and where lockdown measures have been the harshest. This research aims at understanding how the corona crisis has been represented in Spanish and Italian media, focusing on reference newspapers. The study analyzes 72 front pages of El País and El Mundo in Spain and Italy’s Corriere della Sera and La Repubblica, collecting 710 news items and 3456 data evidences employing a mixed method (both qualitative and quantitative) based on content analysis and hemerographic analysis. Results show a predominance of informative journalistic genres (especially brief and news), while the visual framing emerging from the photographic choice, tend to foster humanization through an emotional representation of the pandemic. Politicians are the most represented actors, showing a high degree of politicization of the crisis.

## 1. Introduction

Spain and Italy, with 238,564 and 231,732 confirmed cases, respectively, are amongst the European countries where the COVID-19 pandemic has produced its major impact and where lockdown measures have been the harshest.

Due to the reduced mobility and the imposed lockdown, the internet has proved to play a decisive role in terms of media consumption during the quarantine. Social networks have occupied the first position among online platforms most frequently consulted by citizens. According to Twitter, the information on the pandemic as well as the conversations related to the topic have caused a 23% boost in total active daily users, reaching the general level of 164 million users per trimester. News check-up has experienced a prominent growth at that stage. Specifically, the peak of media consumption coincided with the first measures of social distancing and has increased in correspondence with governmental communications.

These data should be interpreted within the current crisis of journalism and the crisis of media credibility. A recent survey of 27 countries by Ipsos Global Advisor [1] shows how citizens are rather skeptical towards the information they receive from the media, especially when it comes to online press. In Spain, 41% of the surveyed trust in television, whereas 39% expressed their preference for traditional media, such as printed press. Making reference to the intentions, the research claims that half of the total number of the surveyed believe that printed papers have “good intentions” as opposed to 66% considering that online newspapers and web pages are the ones with “the worst intentions” [1].

Based on the trust placed on the printed media—as the most credible and rigorous media—this research analyzes a total of 72 front pages of the main daily newspapers in Spain and Italy (36 each). The research considers the daily newspaper’s front page as a fundamental element that synthetizes and prioritizes the contents that the particular medium treats as the most important. At the same time, the front page maintains a direct relation to the digital version of the medium, somehow setting the agenda. In other words, the front page serves a privileged space for public identity construction [2]. The study, carried out between 24 February and 4 April 2020, collected 710 pandemic-related news pieces and 3456 data evidences, aimed at answering the following research questions:

- How has the COVID-19 pandemic been covered on the front pages of Spain and Italy’s main daily newspapers?
- What types of journalistic genres have been used?
- What types of political or social figures and institutions appear the most?
- What role has been assigned to an image/photograph in the coronavirus-related information items of the front page?

## 2. Theoretical Framework

The COVID-19 crisis has posed new challenges to journalism. Media play a fundamental role in framing a crisis, since providing the right information from a reliable source is the key issue in this type of pandemic. The World Health Organization (WHO) has used the term “infodemic” to define the overabundance of information introduced by coronavirus and to warn the citizens against the risks caused by this information excess, that contain a great amount of hoaxes and rumors. As Sylvie Briand, Director of Infectious Hazards Management at WHO’s Health Emergencies Program notices, this phenomenon is not new “but the difference now with social media is that this phenomenon is amplified, it goes faster and further, like the viruses that travel with people and go faster and further”.

The role of social media in spreading misleading health information is not new [3], but the COVID-19 crisis has shown the critical impact of this new information environment [4]. Many studies have focused on and are still focusing on how the disintermediated role of social media may foster misinformation: Scholars studying Iran [5] and Spain [6], stress how social media spread rumors, others [7] try to analyze the structure of this infodemic, or concentrate on the effect of media exposure [8].

Within this social media euphoria, very few studies have focused on legacy media, intended as the mass media that predominated prior to the Information Age—particularly print media, radio broadcasting, and television—even if reality is showing that legacy media still plays an important role [9].

A study [7] noticed how CNN has recently anticipated a rumor about the possible lockdown of Lombardy (a region in Northern Italy) to prevent the pandemic, publishing the news hours before the official communication from the Italian Prime Minister. As a result, people overcrowded trains and airports to escape from Lombardy toward the southern regions before the lockdown was in place, disrupting the government initiative, aimed to contain the epidemic and potentially increasing contagion. Other literature [10] stresses the importance of looking at mainstream media coverage pointing out the importance of a high quality scientific journalism [11].

The analysis of the printed daily newspapers’ front pages has been object of recurrent studies for the last 50 years. Starting with the classical works [12,13,14,15,16,17,18,19] up to the contemporary researches [20,21,22,23], various studies have dealt with content analysis of that essential element of the printed press [24,25]. Other studies followed them, concentrating on the comparison between the front pages in printed and digital editions of a medium [26].

As previously highlighted, daily newspaper’s front pages are considered to be the most important page, displaying informative priorities and editorial position in relation to current issues [27]. Other studies [28] single out three core elements of a daily newspaper’s front page: Headlines, or visual linguistic set; texts, or visual paralinguistic set and images, or visual non-linguistic set.

In this context, the importance of media and information literacy, seen as the citizens’ ability to access, use, assess, and create responsible and ethical content [29], has become crucial. Media and information literacy refers to the vital role that information and media possess in the everyday life of a person, therefore this skill represents an indispensable component to exercise freedom of expression and information. In this vein, numerous studies [29,30,31,32] stress the significance of a digital literacy development that would exceed studying merely technical or instrumental aspects to embrace the issues of the critical use of media.

## 3. Materials and Methods

The research, based on previous studies [33], analyzes a total of 710 news items extracted from 72 front pages of the four main daily newspapers of Spain and Italy (36 per country). El País and El Mundo of Spain alongside with Corriere della Sera and La Repubblica of Italy were chosen, based on their relevance and the availability of their front pages. The analysis has been carried out through the use of a template chart consisting of 15 parameters and 64 categories that were obtained mainly in inductive form. The study, possessing descriptive and explanatory character, employs a mixed method (both qualitative and quantitative) based on content analysis and complemented by direct observation and hemerographic analysis as the main techniques. The first technique focused on the analysis of various elements that constitute the front page designs by means of a template chart elaborated during the research process. Subsequently, we implemented a hemerographic analysis of texts, headlines and images. The data were processed through descriptive statistics planning with SPSS software. The analytical chart has considered all the elements displayed in Table 1.

## 4. Results

**Figure 1:**
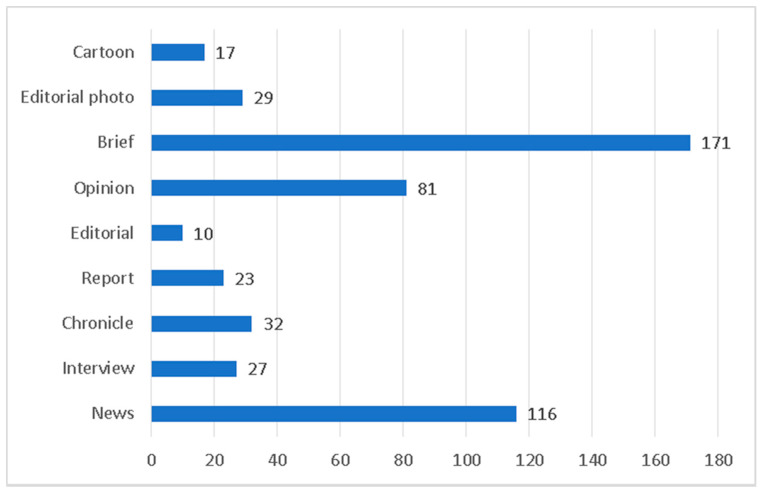
Journalistic genres.

Coronavirus-related information occupies 71% of the front pages. Precisely, 506 news items out of the total 710 focus on topics related to the COVID-19 pandemic. As for the main journalistic genres, (see Figure 1), we can observe brief as the most common. This journalistic genre, characterized by its conciseness and brevity, has been defined as brief, a summarized piece of news that solely reflects the most relevant data of the information, missing profound insight and context. The total of pandemic-related units possess the form of short pieces, that could oscillate between 1 or 5 lines.

News occupies the second position in the list of types of the texts about coronavirus at the analyzed front pages. The informative approach towards, in other words, dominates the representation of the crisis. The effort of the daily newspapers to inform their readers on the characteristics, impact and spread of the virus has been detected. Nonetheless, it is worth mentioning that opinion articles (with a total number of 81 counted units) surpass other informative journalistic genres. Moreover, the importance of the editorial photo with a total number of 38 units, solely accompanied by the footnote, demonstrates a comprehensive approach to the topic through the communicative value of the image.

The location of news items at the daily newspapers’ front pages can be considered another element of high value when it comes to the detection of importance of each topic. In this sense the studies grant more value to the upper part and the right part from the reader’s standpoint. The right part is the most valuable of the odd-numbered page and the left part is the most important for the even-numbered pages. Concretely, projecting an imaginary V onto the open double page, the higher the position on the V, the more value the piece has (both in terms of editorial and advertising rates).

**Figure 2:**
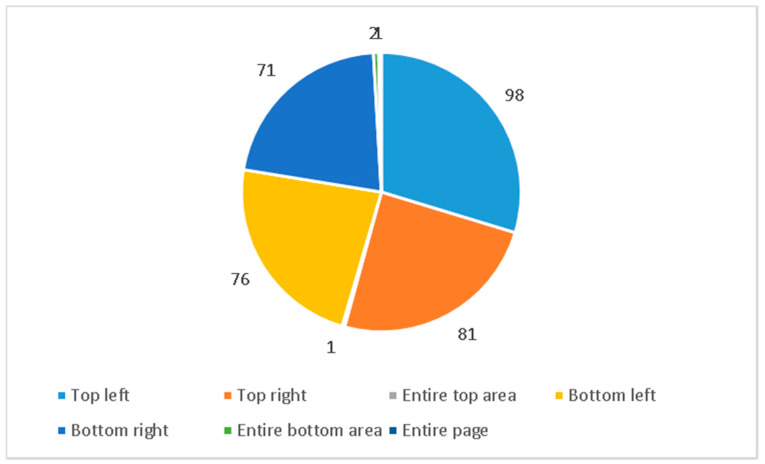
Position on the page.

Accordingly, results show that news about coronavirus appear mostly located in the upper part, but in the left zone (see Figure 2). In this way, it is possible to point out that the newspapers place the news in an important area of their front pages. In addition, in second position, with a total of 81 news items, is the upper right-hand area. In this way, it is possible to point out that the news have been progressively occupying the areas of greatest visual impact of the front page. However, this set of news items is very close to the 147 news items on the pandemic that appear at the bottom of the front page, i.e., the one of least importance. A total of 76 appear at the bottom left and 71 at the bottom right. Therefore, the distribution of the news on COVID-19 between the two areas marked by the horizontal division of the first page (top/bottom) is very tight.

**Figure 3:**
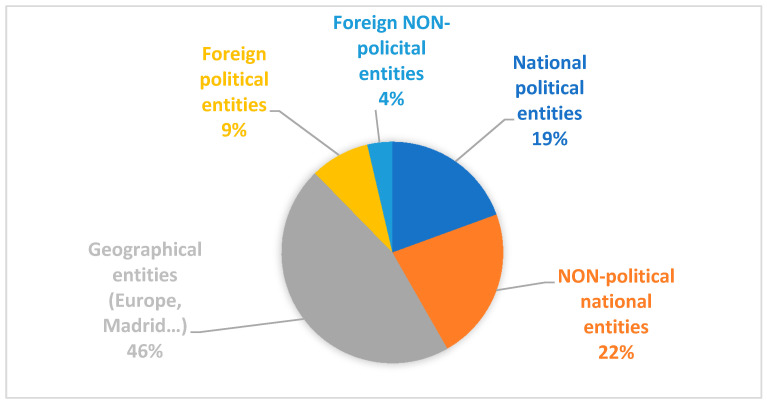
Main entities of the stories.

Figure 3 displays the main entities mentioned in the stories, that is to say institution or entities most recurrently mentioned or displayed. Of the information, 46% mentions geographical scenarios (Europe, Madrid, Milan, etc.). In this sense, there is a tendency to depersonalize the information and to extrapolate it to wider scenarios or territories. This aspect is important insofar as the subject is the element of the sentence that carries out the action contained in it. In 22% of the cases the front page news referred to national entities of non-political nature (the hospital, the emergency unit, the laboratory, the intensive care unit, the sports center, the cultural center, etc.).

In particular, there is a notable reference to entities linked to hospitals and healthcare scenarios. National political entities (government, trade unions, spokespersons, Minister of Health, etc.) occupy the third position in the rank of entities linked to the news, with 19%. Political entities from abroad (the WHO, the European Union, the European Parliament, etc.), with 9%, and non-political entities from abroad (especially universities, research groups or the media), with 4%, respectively, completed the list of entities.

**Figure 4:**
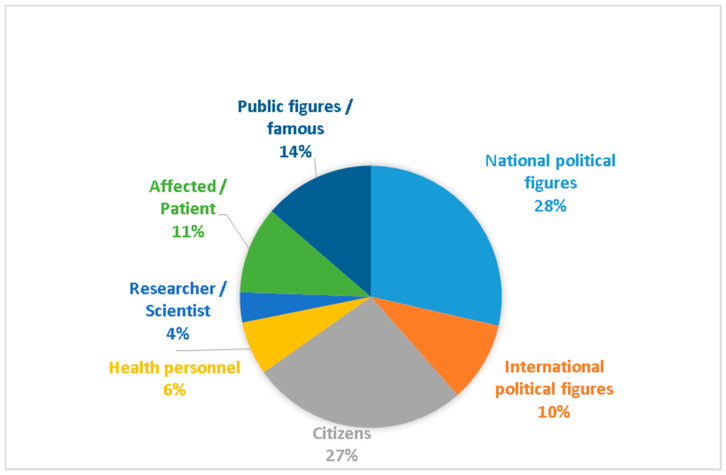
Main characters in the information.

Figure 4 details which kind of people are mostly mentioned within the main characters in the information on COVID-19. Interestingly, national political figures are the most numerous group with 28% of the total, stressing how the crisis is highly politicized. In second place, we find anonymous citizens who are protagonists in 27% of news items on the front page. If political figures normally make it to the front pages, within the COVID-19 crisis, anonymous people have become co- protagonists of the front pages. Public figures from different countries (with 14%) making statements about the pandemic outnumbered those affected by or suffering from the virus (with 11%) and international political figures (with 10%). Finally, health personnel, who have generated important recognition and ovations, have only been the protagonists of 6% of the front page news about the virus, while researchers and scientists (with 4%) occupy the last place in percentage of presence in the front pages.

**Figure 5:**
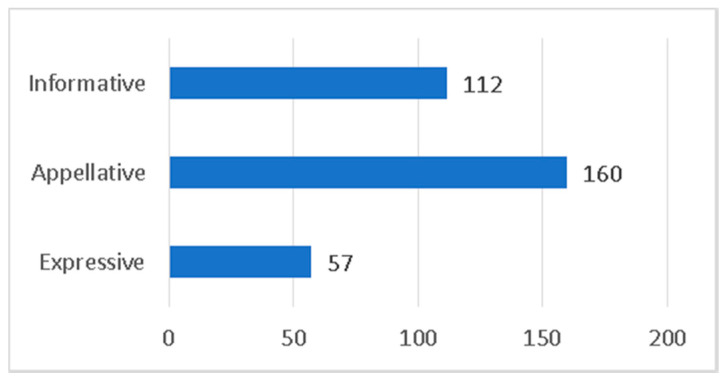
Type of headline.

**Figure 6:**
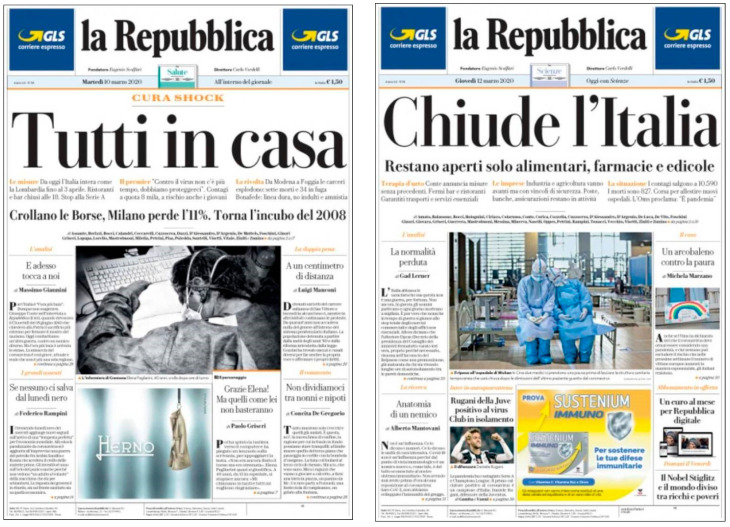
Examples of appellative headlines. Source: *La Repubblica* (2020).

Headlines are also of great importance. Their location on the page, and the type and size of the title contributes to underlining the importance of the information among the set of pieces selected to appear on that page. In relation to this (see Figure 5), the study has identified a predominance (160) of appellative headlines, focused on drawing the reader’s attention. For this reason, the headlines tend to be non-verbal and have very atomized structures that seek to convey to the reader news about a subject he or she already knows. As an example, Figure 6 shows the headlines from Italian La Repubblica: “Tutti a casa” (Everybody is at home) and “Chiude l’Italia” (Italy closes) to announce the lockdown measures.

The continuity of the news about COVID-19 on the front pages of the media, both in Italy and Spain, justifies this tendency towards headlines of an appellative nature that allude to facts that are familiar to the citizens. The informative headlines, with a total of 112 units, are the most conventional and generally opt for the structure of subject, verb and predicate, enunciating a topic related to the pandemic trying to answer the “what” and the “who” of such information. The expressive ones, which have an evocative function on an event known to the reader, are the scarcest (only 57 have been counted). This reduced number emphasizes the existence of a commitment among the media analyzed to avoid sensationalist headlines or those that seek only to externalize moods.

**Figure 7:**
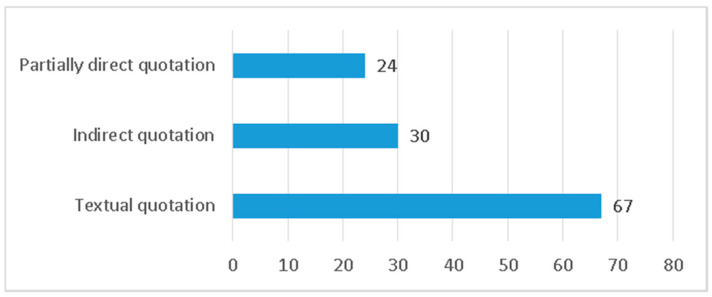
Type of quotation in the headline.

Another classification of headlines focuses on speech acts. In relation to these, as shown by Figure 7, the headlines with textual quotations—which reproduce, between quotation marks, the declaration of one of the protagonists of the information—predominate (67 in total). The majority presence of this type of headlines denotes an interest of the media in presenting the information from a personalized standpoint, thus, bringing the stories closer to the subjects that have generated them. Indirectly quoted headlines (30) and partially direct headlines (24) accumulate a smaller number of cases.

**Figure 8:**
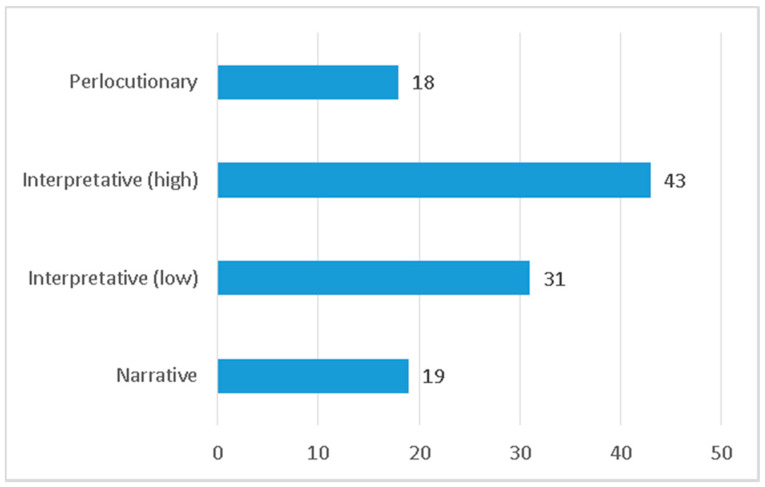
Types of verbs used in the headlines.

By analyzing the types of verbs used (see Figure 8), a predominance of strong interpretative verbs, characterized by highlighting the intensity of an action, is detected (43 in total), followed by weak interpretive verbs (a total of 31) which, although with less intensity, denote a willingness of the journalist to give more intensity to an action. The narrative ones, which are more neutral, add up to a total of 19; while the perlocutionary ones, which incorporate an effect that is intended to be achieved by means of an action, are the least numerous, with 18 units counted.

Out of all the pieces about the COVID-19 crisis, 177, that is to say 35% of the total content on the pandemic, has some kind of photographic accompaniment. Only 2 are in black and white, and in 8 have an artistic quality. The reduced number of photographs on the pandemic on the covers is striking, although it could be justified by the difficulty of obtaining images or doing so from a variety of themes that would allow for dynamism and renovation of the covers.

Regarding the characters that appear in the photographs, public figures such as Pope Francis, or celebrities, like sportsmen or writers have taken over the front pages (28.85%). The second leading role is played by anonymous citizens who appear in everyday scenes, with a total of 24%, and national politicians account for 19%. Only 3% have images of people affected by or patients who have contracted coronavirus. International political figures account for 8% of the total. Finally, health personnel only appear in 14% of the photographs and scientists and researchers in 3%.

**Figure 9:**
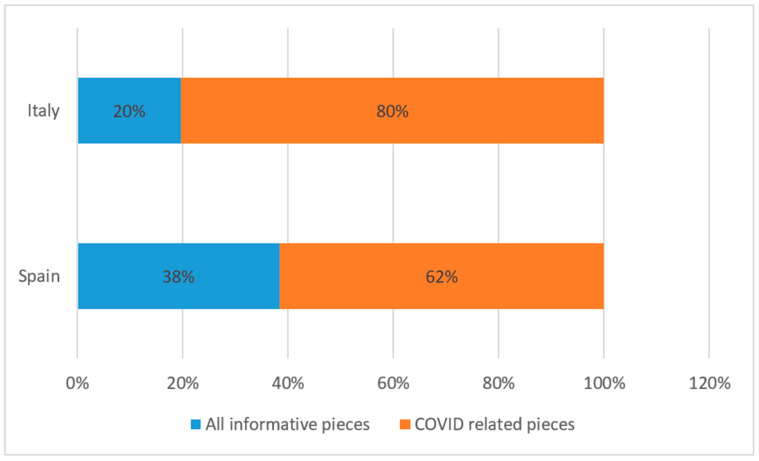
Total number of information pieces in Spain and Italy.

The visual framing also shows a certain level of both spectacularization (with the presence of celebrities) and politicization of the crisis, while health workers and scientists that actually are on the frontline of the fight against the virus are less visible. By comparing Italian and Spanish news outlets, we can observe how COVID-19 occupies the majority of the information in both countries. Nonetheless, while in Spain it occupies 62% of the front page; in Italy COVID-19 related pieces cover a striking 80% of the information (see Figure 9). Italy was the first European country severely hit by the pandemic, so it makes sense to state that this unpleasant surprise somehow engulfed media attention.

With regard to the information that presents a predominance of numeric data, the number of pieces is very low in both countries. Spain, with 9%, and Italy, with 2%, reinforce the scarcity of information focused only on figures or percentages. This might seem surprising, due to the overwhelming amount of data information (statistics, evolution of case numbers, etc.) we have received during the pandemic, nonetheless it confirms the interpretative role assumed by the printed press: While on line media can offer on line updating, the printed press offers a more interpretative vision of facts.

**Figure 10:**
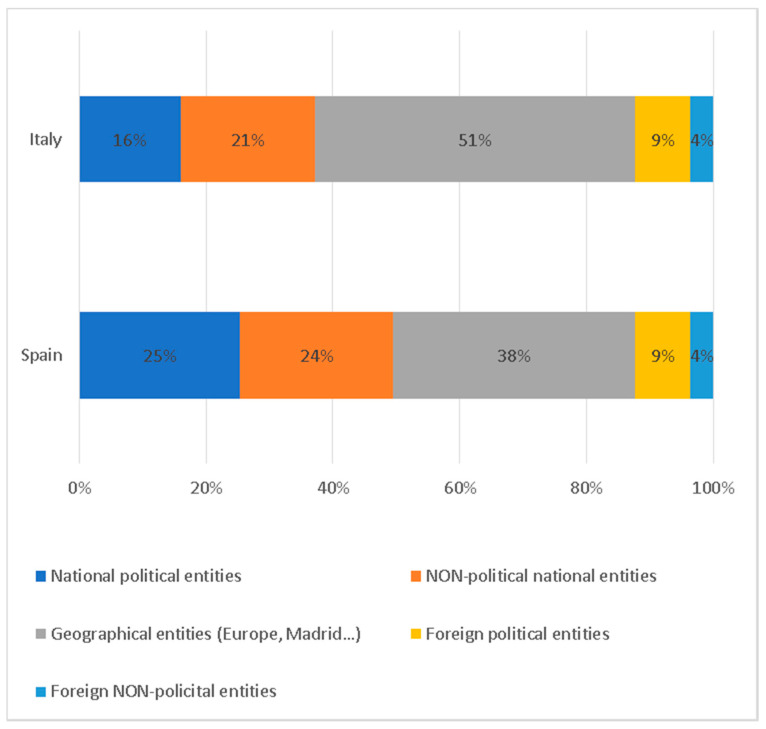
Type of entities/institutions represented in the information.

Figure 10 displays the main entities portrayed in the information. Geographical names, that is to say cities or regions, are the most numerous in the information on COVID-19 (51% in Italy and 38% in Spain), followed by national institutions not linked to politics, which, with 21% in Italy and 24% in Spain, show the prominence that this type of institution has acquired in the framework of this crisis. Political institutions are those that occupy the third place with 16% of the total in Italy and 25% in Spain.

**Figure 11:**
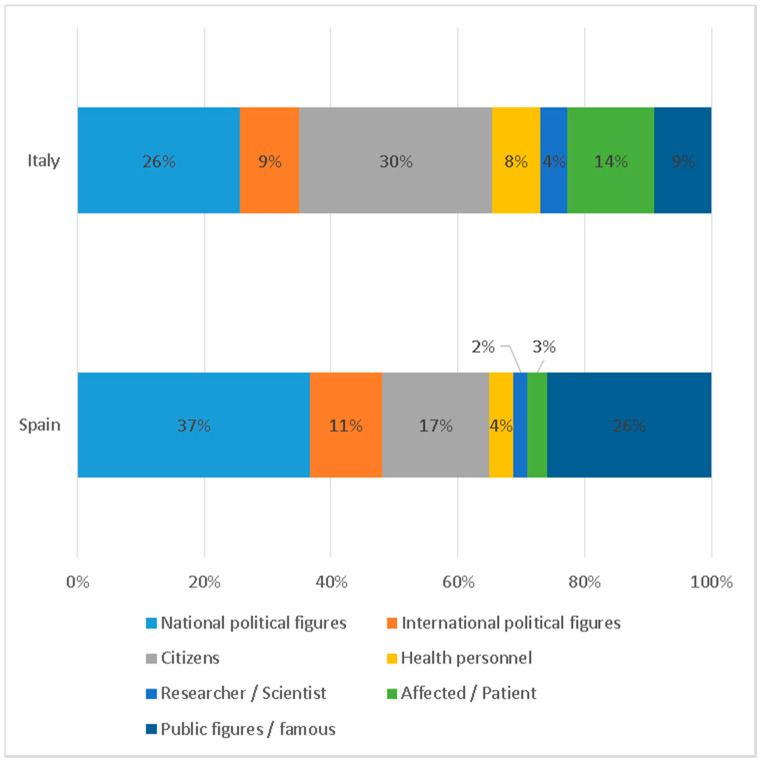
Main characters of the information.

The characters that appear in the information correspond to very diverse profiles. National political figures are the most numerous, with 26% in Italy and 37% in Spain (see Figure 11). This aspect contrasts with the reduced presence of institutions, as mentioned above. Thus, it is possible to point out that politics is personalized as party politics through the representation of figures of the different parties of the country that complies with the seminal findings of Hallin and Mancini [41].

In Italy, citizens account for 30% of the total number of items; in Spain, they account for only 17%. These differences are equally visible in the presence of researchers or scientists, which in Italy is 9% and in Spain reaches 26%.

**Figure 12:**
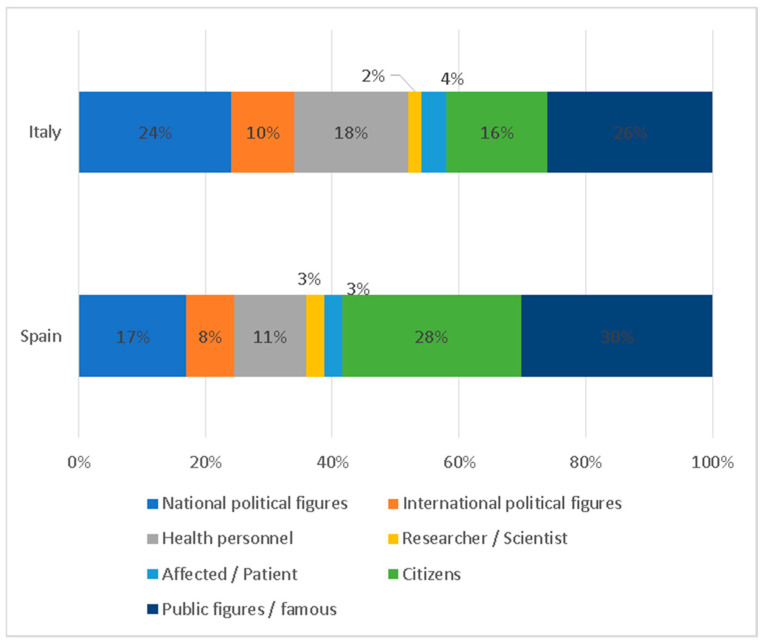
Main characters displayed in the pictures.

The main characters that appear in the photographs on the covers, displayed by Figure 12, show important differences between the two countries. Celebrities or public figures are the ones that absorb the most attention, with 26% of the total in Italy and 30% in Spain. This aspect emphasizes the importance given to this type of profile in the information and awareness of the pandemic. Citizens, with 16% in Italy and 28% in Spain, would be in second place. There is, therefore, a prominent role for anonymous people. National politicians, with 24% and 17%, respectively, would be in third place. Patients (with 4% and 3%) and researchers or scientists (with 2% and 3%) hardly appear in the cover images.

**Figure 13:**
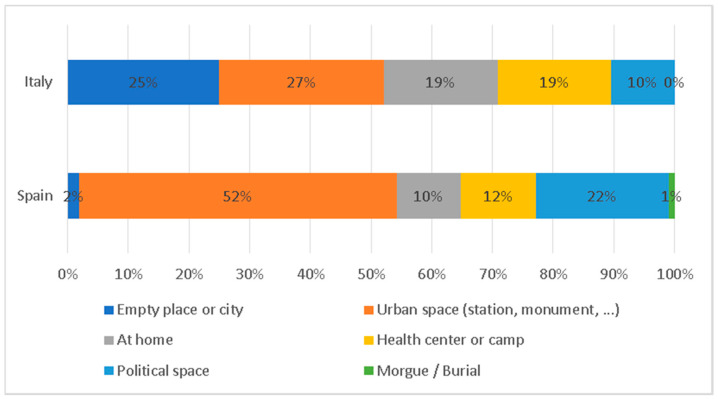
Spaces portrayed in the pictures.

Looking at the physical spaces represented in the news, summed up in Figure 13, we can observe similarities and differences. First, while Italian newspapers offer an emotional representation of the crisis by granting an enormous importance to the representation of empty spaces (such as squares or symbolical touristic spots, like the Trevi fountain in Rome, that in a normal situation would be crowded), Spanish news outlets completely avoid this option, that only account for 2% of the total pictures. In both cases, however, urban spaces are the most recurrent (with 27% in Italy and 52% in Spain). In addition, although their importance is not prominent, the spaces related to political life (Congress, etc.) with 10% in Italy and 22% in Spain, have a significant presence in the cover photographs. Health centers or health camps, with 19% and 12%, are other places that appear next to citizens’ homes (with 19% and 10%, respectively).

**Figure 14:**
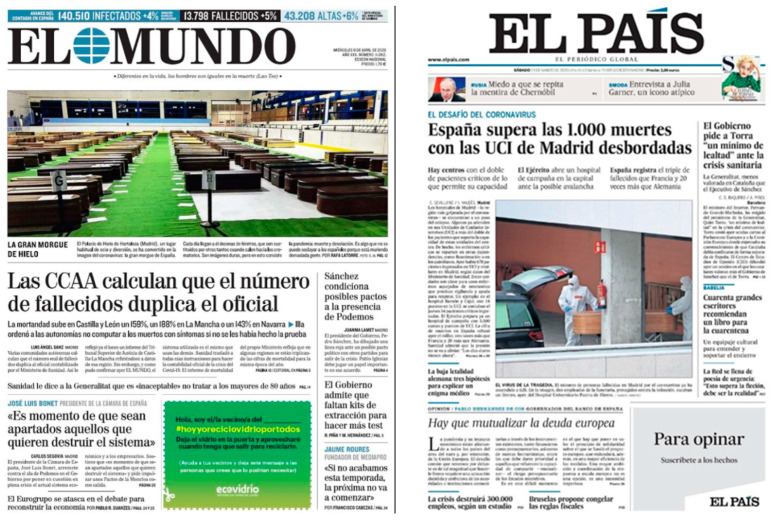
Examples of pictures. Source: *El Mundo* (2020) and *El País* (2020).

Even if health centers are not the most prominent settings in pictures, we observed how, when portrayed, these spaces are emotionally charged to dramatize the tragedy. Figure 14 shows two examples of Spanish newspapers El Mundo y El País showing coffins of victims in the middle of the crisis.

## 5. Discussion

The COVID-19 crisis has been a shocking reality that took most countries by surprise. Italy and Spain have been amongst the first in Europe to be hit by the pandemic. Thus, observing media behavior is especially interesting. To answer our research questions, we can state that the COVID-19 crisis has been covered mainly in an informative way: The analysis of the two main newspapers in Spain and Italy allows to observe a predominance of informative journalistic genres. In particular, the predominant genre is the brief, short news items, which lack contextual information, and do not offer in depth information to the readership.

However, the choice of images, that is to say the visual framing of the stories, seems to suggest an emotional turn. In other words, even if the predominance of informative genres, together with the avoidance of openly emotional headlines might suggest that the analyzed newspapers have avoided sensationalism, both the mentioned visual framing and the increased presence of anonymous citizens and celebrities among the subjects, can be interpreted as an attempt to humanize the information pieces, emotionally charging them. In particular, it is important to stress out the high level of politicization of the crisis: Politicians have been the most recurrent actors both in the information and in the pictures.

This, as seen, seems contradictory to the scarce presence of institutions. This result, however, should not be of surprise since, as already pointed out by Hallin and Mancini [41], both Spain and Italy belong to the polarized pluralist model, in which party politics is predominant to institutional politics. Moreover, both countries are characterized by high intensity political polarization, therefore the management of the crisis has been the source of harsh political conflicts between government and opposition, that has been reflected by the media. Concretely, Spanish media outlets are the ones that give a more political vision of the crisis.

Accordingly, we observed a trend to objectify the different news actions and events through the use of geographical entities. The use of physical enclaves (Italy, Spain, Milan or Madrid, for example) lead to a simplification or generalization of reality that can bias the reading and interpretation of what has happened.

In the same vein, it is important to stress that both health personnel and researchers directly involved in the fight against the virus, have a negligible presence both in pictures and information.

In conclusion, we can sum up that the protagonists of the pandemic are not those affected, or involved in the fight, but rather anonymous citizens, and especially celebrities and politicians.

These results cannot be discussed without taking into consideration the general framework of the social responsibility theory. As pointed out by McQuail in his seminal work [42], media should accept and fulfil certain obligations to the society and should meet high professional standards of accuracy, truth, objectivity and balance. Therefore, journalists and professionals should be accountable to the society reflecting and respecting diversity, pluralism as well as diverse points of view and rights of reply.

Applying these criteria to the specific field of health communication, defined by Sixsmith et al. [43] as the study and use of communication strategies to inform and influence individual and community decision that enhance health, encompassing health promotion, health protection, disease prevention and treatment, we see how media are pivotal to the overall achievement of the objectives and aims of public health.

In this sense, media practitioners and their organizations should be in charge of delivering rigorous health information, aimed at creating awareness about people’s health, to prevent diseases and encourage healthy living.

One of the main requirements of good health journalism [44], thus, is to present evidence-based news with proper perspective, and without giving rise to sensationalism or alarm.

As pointed out by many studies [45,46] journalism training, in order to cope with these new challenges, should lay special emphasis on these aspects, providing not only specific health journalism training, but developing specific media and information literacy training devoted to health issues.

## 6. Conclusions

Acknowledging the geographical limitations, our study allows a series of conclusions to be drawn, which, from a diagnostic perspective, may help both scholars and journalism practitioners and deepen on the behavior and reaction capacity of newspapers in front of important and tragic events such as planetary pandemic.

From a scholarly perspective, our work is embedded in a stream of literature that considers media to play a crucial role in framing public debates and shaping public perceptions by selecting which issues are reported and how they are represented [20,21,22,23].

Even if, as said, our results are limited to Spain and Italy, we have shown that printed newspapers avoid the massive use of data or percentages, leaving live updates to on line media, concentrating on more informative and interpretative pieces. This, on the one hand suggest they still play a crucial role in molding public opinion by offering more interpretative content [9], on the other they directly and indirectly interact with digital media, in charge of giving more live information. For this reason, our results suggest that legacy media should still be examined to see how they influence/are influenced in their interaction with online media.

In addition, we have pointed how in both countries the pandemic has been highly politicized. This result, stressing out the salience of the political factor in representing the pandemic, underlines the need for more comparative research, analyzing media portrayal in different contexts and how different media, embedded in different political and cultural contexts, have reacted.

In particular, the current corona crisis, having a global reach and effect, could be an ideal occasion to compare media behavior in different countries to observe the existence of similarities and differences, and to which extent different political cultures and political systems modify media reactions to a pandemic, following and proving Hallin and Mancini’s model [41].

In addition, even if reference newspapers seem to opt for an informative approach, their visual framing and the choice of images (i.e., empty places) emotionally charges the information. In this sense, besides the need for more comparative research to prove that this is a global trend, from a practical standpoint, our results align with the findings of previous studies [45,46], stressing the need to promote media and information literacy, not only among citizens, but also among media professionals. As demonstrated, both the predominance of short news items, which lack contextual information, and the visual framing can make the process of processing the essence of information messages difficult, making the susceptibility of many people to misinformation as risky as susceptibility to the virus itself.

Thus, as previously pointed out, in order to achieve rigorous and responsible health information, journalism training should not only provide specific health journalism training, rather media and information literacy skills should be developed in this field.

In the same vein, media and information literacy campaigns geared towards citizenship should focus specifically on health issues, since, as the current crisis has showed, health is one of the most sensitive topic when it comes to quality information to avoid the risk of misinformation.

Accordingly, the current corona crisis underlines the necessity of a reform of science communication. This pandemic has underlined that the media often does not offer rigorous scientific information, prioritizing a (possibly) misleading humanization of news.

First, media professionals should be trained and help to implement fact-checking functions, particularly to debunk fake news, misinformation and disinformation on health subjects.

Moreover, a renewed cooperation and a greater communication between media, health experts, academia and policy makers is essential for improving quality of health news. For this, academic institutions, health bodies, and organizations engaged in scientific and medical research need to improve their communication with media, understanding the need to explain research findings, policies and trends to media professionals, who are in charge of ”translating” and diffusing them to citizens. On the other side, media should rely on competent scholars from a wide range of disciplines, interacting, assessing and dialoguing with journalists in order to provide readers, and ultimately citizenship, with a better understanding of science-related issues such as a pandemic.

## Figures and Tables

**Table 1:** Elements of the analysis.

Items at the Front Page	Total Number
Coronavirus-Related Items	Total Number
Journalistic genre	News, interview, chronicle, report, editorial, opinion article, brief, editorial photo, cartoon
Location on the front page	Top left, top right, entire top area, bottom left, bottom right, entire bottom area, entire page
Type of prevailing information	Data or interpretative
Entities/institutions in the information	National non-political entities, national political entities, geographical entities, international/foreign political entities, international/foreign non-political entities, others
Main characters in the information	Home politicians, international/foreign politicians, citizens, doctors and medical staff, scholars-researchers, patients and the affected, famous people/celebrities and others
Type of the headline	Informative, expressive or appellative
Type of quotation in the headlines	Direct quote, indirect quote or partially direct quote
Type of verbs in the headline	Narrative, interpretative weak, interpretative strong or perlocutionary
Images/photos	Number of photos/images
Type of the image/photo	Color or black and white
Function of the image/photo	Documentary or artistic
Main characters of the image/photo	Home politicians, international/foreign politicians, citizens, doctors and medical staff, scholars-researchers, patients and the affected, famous people/celebrities and others

Source: Elaborated by the authors based on [34,35,36,37,38,39,40].
